# Chinese pediatric *Tuina* on children with acute diarrhea: study protocol for a randomized sham-controlled trial

**DOI:** 10.1186/s13063-019-3818-1

**Published:** 2019-12-09

**Authors:** Taoying Lu, Huiyan Zhang, Lingjia Yin, Jianxiong Cai, Meiling Li, Lin Dai, Conghao Zhu, Yongping Zhang, Feng Xiang, Li Wang, Lu Li, Lixin Wang, Darong Wu

**Affiliations:** 1 Program for Outcome Assessment in TCM, 2nd Affiliated Hospital of Guangzhou University of Chinese Medicine, Guangzhou, China; 2 Health Construction Administration Center, Guangdong Provincial Hospital of Chinese Medicine, Guangzhou, China; 3 Guangzhou University of Chinese Medicine, Guangzhou, China; 4 Gastroenterology Department, Guangzhou Hospital of TCM, Guangzhou, China; 5 Acupuncture and Tuina Department, Wenzhou Hospital of Chinese medicine, Wenzhou, China; 6 Pediatric Department, Dongguan Kanghua hospital, Dongguan, China; 7 Department of Tuina, Affiliated Hospital of Changchun University of Chinese Medicine, Changchun, China

**Keywords:** Acute diarrhea, Pediatric *Tuina*, Randomized controlled trial

## Abstract

**Background:**

Acute pediatric diarrhea is one of the most common causes of morbidity and mortality worldwide and seriously affects the health of children. Previous studies have shown that pediatric *Tuina*, a traditional Chinese medicine therapy, has potential therapeutic benefits for acute pediatric diarrhea. However, the evidence for its effectiveness is insufficient due to the lack of high-quality clinical studies. Our aim is to evaluate the efficacy of Chinese pediatric *Tuina* for children aged 0–6 years with acute diarrhea.

**Methods/design:**

This study is a randomized, double-blind, sham-controlled trial. We will include 122 children with acute diarrhea from Dongguan Kanghua Hospital in Guangdong province, China. The patients will be allocated into either the pediatric *Tuina* group or the sham *Tuina* group in a 1:1 ratio. The treatment will last for 3 days followed by an 11-day follow-up period. Both groups will receive usual care. In addition, the experimental group will receive 15–25 min of Chinese pediatric *Tuina*, while the control group will receive 15–25 min of sham pediatric *Tuina.* Both groups will receive treatments once per day, for 3 consecutive days. Primary outcome measures are diarrhea days from baseline and diarrhea times on the third day. Secondary outcome measures are the global change rating and period of days when the stool character changes to normal. Safety assessments will be monitored during each visit.

**Discussion:**

This clinical trial is designed to evaluate the efficacy of pediatric *Tuina* for children with acute diarrhea. We expect results to provide solid evidence and support for pediatric *Tuina* as an appropriate treatment for children with acute diarrhea.

**Trial registration:**

ClinicalTrials.gov, NCT03005821. Registered on 29 December 2016.

## Background

Acute pediatric diarrhea is a common and potentially serious condition with global significance. It is a major cause of morbidity and mortality for children, especially in low- and middle-income countries [1, 2]. In 2010, there were more than 1.7 billion diarrheal episodes in children under 5 years of age. Among these, about 36 million cases progressed to severe episodes, accounting for over 700,000 deaths annually [3]. Although the number of deaths due to diarrhea fell to 499,000 globally in 2015, diarrhea remains one of the leading causes of death among children younger than 5 years [4]. While the burden is greatest in low-income populations, acute diarrhea is also a common reason for outpatient visits and hospital admissions in high-income regions and has become an important global health problem [5]. Considering the severity of pediatric diarrhea, if patients are treated in an untimely manner then repeated episodes of diarrhea can lead to undernutrition and stunted growth and can further decrease cognitive function [3]. Therefore, research on childhood diarrhea has become a priority of the World Health Organization (WHO) for achieving the United Nations’ Millennium Development Goal of reducing childhood mortality [6].

The recommended treatments for diarrhea include continued feeding or increased breastfeeding, low-osmolarity oral rehydration solution (ORS) or intravenous infusion, probiotics, zinc supplementation and antimicrobial usage for bloody diarrhea [6–9]. These therapeutic methods play important roles in the treatment of diarrhea; however, some interventions, such as zinc supplementation and ORS, are not easily accepted by children or their parents which can lead to low compliance and consequently impacting their effect [10–12]. Studies have shown that ORS takes a relatively long time to produce positive outcomes and may increase the risk of diarrhea volume [13, 14]. Additionally, antimicrobials are not recommended for routine use as overuse causes intestinal flora imbalance, drug resistance, adverse reactions and increased financial burden [15]. More effective and targeted antidiarrhea treatment methods are needed.

Pediatric *Tuina* is a form of traditional Chinese medicine (TCM) therapy in which trained practitioners manually stimulate specific acupoints of the body which are located primarily on the fingers, palms, arms, head, abdomen and back, and include points such as *bagua, banmen* and *zhongwan* [16]. It is based on TCM *zang-fu organ* theory and meridian theory and seeks to unblock meridians, promote the circulation of *qi* and blood, regulate the functions of the *zang-fu* organs, and strengthen the body’s resistance to pathogens by using various manual techniques at specified locations on the surface of the body [17]. *Tuina* manipulations include pushing, kneading, grasping, pounding, arc-pushing, and so forth. All manipulations are conducted with a light, fast, and soft touch [16].

Some randomized controlled trials (RCTs) for children with acute diarrhea have suggested a benefit from pediatric *Tuina* with few side effects [18–21]. However, the methodological quality of some RCTs is poor which may weaken the validity of their results [22, 23]. Lack of scientific rigor or external validity limits widespread application of *Tuina*. The aim of this study is to evaluate the efficacy of pediatric *Tuina* as an add-on therapy compared with sham *Tuina* in addition to usual care for children aged 0–6 years with acute diarrhea.

The study protocol was written in line with the Standard Protocol Items Recommendations for Interventional Trials (SPIRIT) 2013 statement [24]. Details are provided in Additional file 1.

## Methods/design

### Study design

**Fig. 1:**
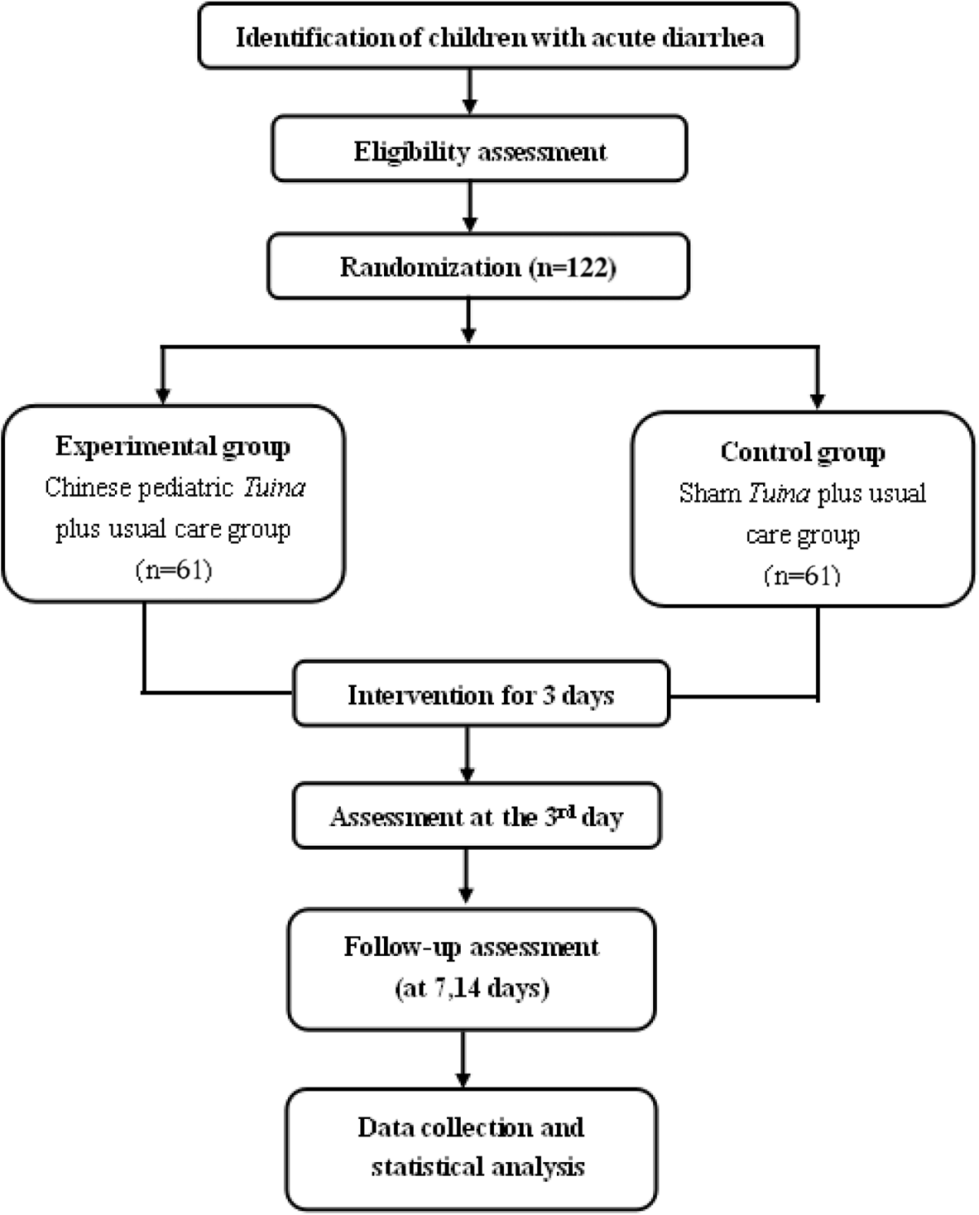
Flow chart of the trial

The study is a randomized, double-blind, sham-controlled design. The trial will be conducted from January 2017 to September 2019 in Guangdong Provincial Hospital of Chinese Medicine and Dongguan Kanghua Hospital in Guangdong province, China. This trial will include a 3-day continuous treatment period and a follow-up period with two visits (on days 7 and 14). Eligible participants will be randomly assigned to either a pediatric *Tuina* plus usual care group or a sham *Tuina* plus usual care group in a 1:1 ratio according to a computer-generated randomized table. Figure 1 shows the flow chart of the trial. The findings of the trial will be reported in accordance with the Consolidated Standards of Reporting Statement [25] and Standards for Reporting Interventions in Clinical Trials of Acupuncture guidelines [26]. The trial has been registered at ClinicalTrials.gov (NCT03005821).

### Participants

The participants will be recruited from Dongguan Kanghua Hospital in Guangdong province, China. All participants will complete a screening form to assess their eligibility. This study will enroll 122 children with acute diarrhea. The diagnosis of diarrhea will be based on criteria as identified by Shen and Wang [27] and the WHO [28].

### Eligibility criteria

#### Inclusion criteria

Inclusion criteria are as follows:

1. Children aged 0–6 years
2. The first occurrence of diarrhea within 72 h
3. Children who meet the acute diarrhea diagnostic criteria identified by Shen and Wang [27] and the WHO [28]
4. Stool frequency greater than or equal to five times per day
5. No concurrent involvement in any other clinical trial
6. Guardian or parents able to coordinate care during the clinical trial
7. Written informed consent by the children’s guardian or parents

#### Exclusion criteria

Exclusion criteria are as follows:

1. A history of Chinese pediatric *Tuina* on the hands
2. Diagnosis of cholera or malaria
3. Children with any of the following conditions on the area to be manipulated: phlebitis, open wound, fracture, or tissue damage
4. Children with any of the following complications: severe dehydration, metabolic acidosis, disorders of consciousness, seizures, twitching, shock, or azotemia

### Interventions

#### Experimental group

Patients will receive montmorillonite powder and racecadotril granules as usual care according to clinical symptoms. In addition, intravenous infusion, ORS, zinc or antibiotics will be used if the pediatrician in charge considers it necessary. Dosage will be prescribed according to the age or weight of the child.

In addition to usual care, the experimental group will also receive Chinese pediatric *Tuina* treatment for 15–25 min, once per day, for 3 consecutive days. *Tuina* manipulation will be performed by trained therapists to ensure identical *Tuina* treatment according to standard operating procedures. Treatment will be performed with the child sitting either on a bed or on a stool. Children under 2 years of age will sit on their caregiver’s lap while receiving therapy. Moderate pressure *Tuina* therapy will be provided. The child will be draped with a cloak that can cover the child’s upper body, including the upper limbs and trunk, and all manipulations will be performed under the cloak.

The standardized *Tuina* therapy prescription includes six acupoints suggested by a panel of five pediatric *Tuina* experts who have a minimum of 10 years of clinical experience in this area. Manipulation times for each acupoint will depend on age. Detailed information for acupoints, manipulation times and methods is provided in Table 1.

**Table 1:** *Tuina* acupoints, manipulation times and methods

Age	Total times					
	*Neibagua* point-arc-push, clockwise	Small intestine meridian point-clearing	Large intestine meridian point-nourishing	*Banmen* point-kneading	Abdomen-rubbing, anticlockwise	*Qijiegu* point- pushing up
<2 years old (0–1 years old)	500	300	300	300	300	300
≥2 years old and <4 years old (2–3 years old)	700	500	500	500	500	500
≥4 years old and ≤6 years old (4–6 years old)	1000	800	800	800	800	800

#### Control group

Patients in the control group will receive the same usual care as the experimental group. In addition, the control group will receive sham pediatric *Tuina*. The therapist will use one hand to hold the child’s hand or put one hand on the child’s body while the other hand performs manipulations on the therapist’s own hand instead of the child’s hand or body. Patients will be draped with the same type of cloak as in the experimental group, and all manipulations will be performed under the cloak. Manipulation times for each acupoint will also depend on age. Sham *Tuina* will be performed for 15–25 min, once per day, for 3 consecutive days.

### Outcome assessment

Two assessors who are blind to group allocations will perform outcome assessment.

#### Primary outcomes

The primary outcome measure for diarrhea will be diarrhea days from baseline and diarrhea times on the third day. The assessment of diarrhea days refers to the period of days from baseline to the first day that diarrhea is reduced to less than or equal to two times per day throughout the treatment and follow-up period. Diarrhea times will be evaluated on the third day of intervention. In this study, we define the baseline and the first day of intervention as the same day.

#### Secondary outcomes

Secondary outcome measures include both a global change rating (GCR) and the period of days when the stool character returns to normal. The GCR will be evaluated on days 7 and 14 after baseline and describes a patient’s overall clinical state as a “global impression”, as determined by the assessor. The GCR will be based on a five-point scale as follows: much better, slightly better, unchanged, slightly worse, and much worse. The period of days when the stool character returns to normal is the number of days from baseline to the first day that the stool character returns to normal throughout the treatment and follow-up period.

#### Safety assessment

The incidence of treatment-emergent adverse events (AEs) will be monitored throughout the entire *Tuina* treatment. Common AEs include local skin damage and rash. Should an AE occur during treatment, the time of occurrence, severity, progress, and treatment of the AE will be recorded in detail. If any serious AE occurs the Principal Investigator and the Institutional Review Board will be informed immediately and direct action will be taken immediately.

#### Unexpected event processing

In the event of damage to the skin, manipulations shall immediately cease. If necessary, affected areas will be treated with an anti-inflammatory cream. For severe cases, antibiotics will be used to prevent infection.

In the event of skin redness or a rash occurring, manipulations shall immediately cease. For severe skin rashes, treatment will be as provided as deemed appropriate by a pediatrician.

### Participant timeline

**Fig. 2:**
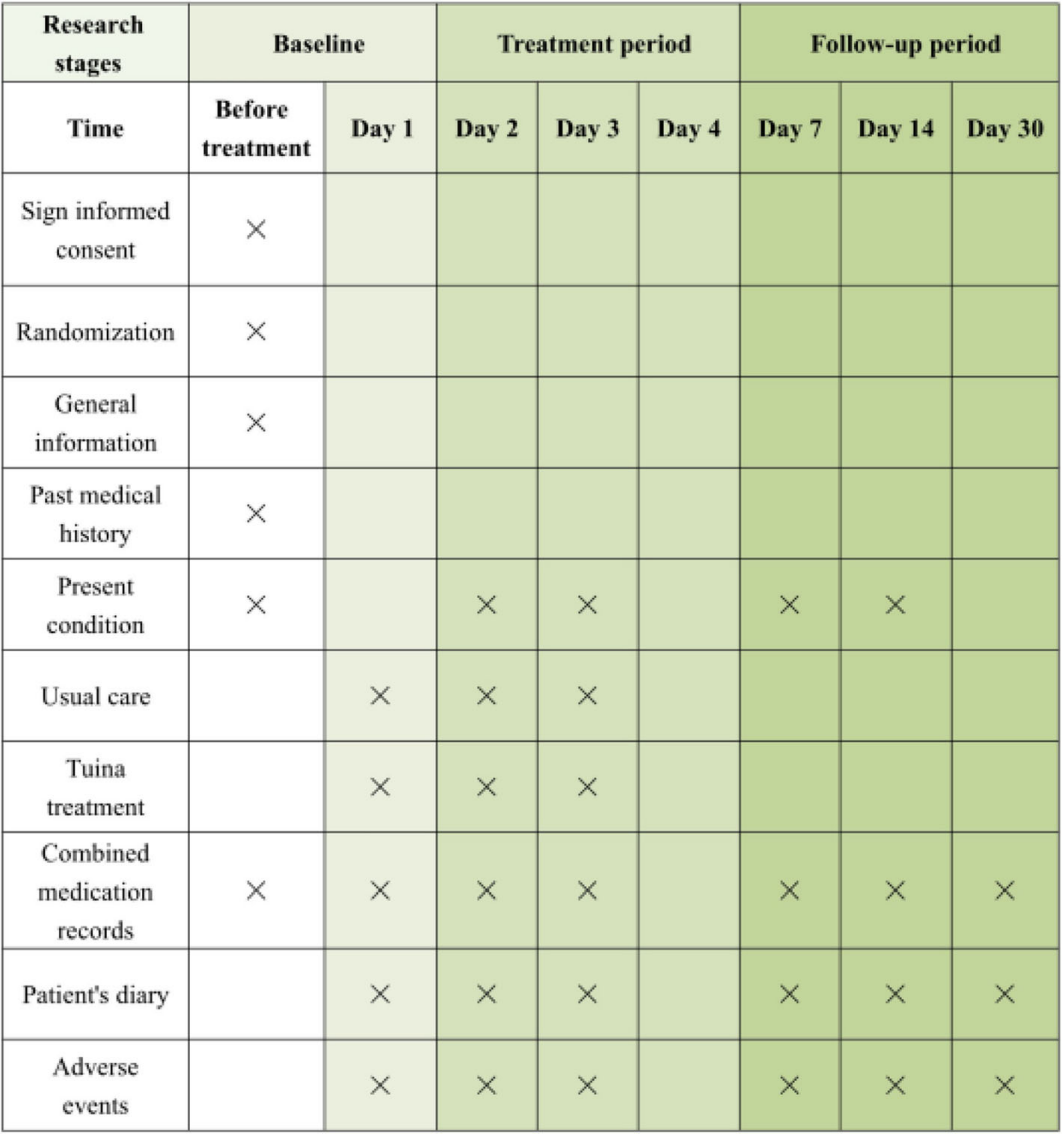
Study schedule for recruitment, interventions, outcome measurements and data collection

Figure 2 demonstrates the overall schedule for participant recruitment, intervention, assessment and follow-up, based on the SPIRIT figure.

### Sample size estimation

This study includes two primary outcomes: diarrhea days and diarrhea times. Sample size was estimated based on the two primary outcome measures. Estimation according to diarrhea days was based on results from previous relevant literature [29], which established diarrhea days in children as 2.1 days after receiving *Tuina* plus usual care. The estimated standard deviation is 1.4. Diarrhea days in children after receiving usual care is 4.0 and the estimated standard deviation is 3.5. On the basis of a 5% false-positive error rate (α = 0.05, two-sided) and 90% power (β = 0.1), sample size was estimated using PASS11 software. A sample size of at least 35 participants should be recruited in each group. Assuming an approximate 20% dropout rate, a total of 84 (70 plus 20% is 84) participants should be enrolled in the trial (42 in each group).

Estimation according to diarrhea times was likewise based on reference from previous relevant studies [30], which established diarrhea times in children as 4.1 times per day after receiving *Tuina* plus usual care. The estimated standard deviation is 1.5. Diarrhea times of children after receiving usual care is 6.2 times per day and the estimated standard deviation is 4.8. On the basis of a 5% false-positive error rate (α = 0.05, two-sided) and 90% power (β = 0.1), sample size was estimated using PASS11. A sample size of at least 51 participants should be recruited in each group. Assuming an approximate 20% dropout rate, a total of 122 (102 plus 20% is 122) participants should be enrolled in the trial (61 in each group).

Based on these results, we chose the larger number sample size, that is 122 participants, for the estimated final trial sample size.

### Recruitment

Several strategies will be applied to recruit participants. In this RCT, we will recruit participants both from the pediatric inpatient and outpatient departments of Dongguan Kanghua Hospital. We will advertise in public areas of the hospital and through media such as WeChat (a Chinese multipurpose social media mobile application software), which will provide brief descriptions of the eligible population as well as the purpose, procedures, interventions, possible risks of the trial, and contact information for the researchers. After eligibility assessment, the guardians of all eligible patients will be requested to sign informed consent forms. Clinical recruitment staff will be responsible for the recruitment process.

### Randomization and allocation concealment

Eligible patients will be allocated by research staff according to a computer-generated random sequence in a 1:1 ratio to the two groups (*Tuina* or sham *Tuina*) of the study. Random allocation will be generated by means of sealed envelopes by the independent statistician and will not available to any member of the research team until the databases are completed and locked. According to the random number table, allocations to the two groups will be assigned and coded by persons who are not involved in the clinical trial and will then be placed in sealed opaque envelopes with sequence numbers also containing the group assignment and type of intervention. When new participants are enrolled in the study, the *Tuina* therapists will open the envelopes in sequence and assign participants to the relevant intervention accordingly. All processes will be recorded and saved as appropriate.

### Blinding

In this study, patients, caregivers, research staff, outcome assessors and data statisticians will be masked to assignment. However, *Tuina* therapists cannot be masked as sham *Tuina* will be practiced differently from real *Tuina*. Participants will be assigned to receive their corresponding interventions in separate rooms to avoid communication. *Tuina* therapists will not be allowed to share any information in terms of patient intervention with other researchers, including outcome assessors and data statisticians. Furthermore, therapists are prohibited from speaking with guardians or participants about group allocation information. To ensure successful blinding implementation, therapists will perform manipulations under an opaque cloak-shaped device, big enough to cover the upper body and arms of a child who is 6 years old or younger. Two narrow holes are present for therapists to insert their hands through and perform *Tuina*. The entire manipulation process will be covered by the cloak. In addition, the outcome assessors who are not aware of what kind of treatment the children received will ask the participant’s guardian to complete the questionnaire independently in a separate room during the intervention as well as the follow-up period and will not be allowed to directly communicate with the therapists during the whole study process.

### Data collection

In this study, two data collectors will be responsible for collecting all data when the children are recruited. A printed case report form (CRF) with a three-digit identification number for each participant and electronic follow-up questionnaires will be used to record all information. Each participant will have a unique three-digit identification number that will be pre-recorded on the printed CRF. The social demographic characteristics of the participants including age, height, weight, medication history and present condition will be collected. Clinical outcomes will also be collected by outcome measurement instruments during the entire intervention and follow-up period. Participants will be appropriately compensated if they complete the entire procedure. During the intervention and follow-up period, electronic questionnaires will be sent to the participant’s guardian through WeChat. For those guardians who do not have a WeChat account, telephone surveys will be used to fill in printed questionnaires which contain the same information as the electronic version. All electronic information obtained through WeChat will be recorded on reserved sections in the CRF. AEs, if any, will also be recorded. Participants have the right to withdraw from the study at any time, for any reason.

### Data management

CRFs completed in Dongguan Kanghua Hospital will be sent to the research office based in the 2nd Affiliated Hospital of Guangzhou University of Chinese Medicine. A data administrator will be responsible for checking the completeness and logicality of the data. Afterwards, double entry will be performed independently by two team members, and accuracy will be checked using point-by-point comparison with the original data. The data manager and the trial statisticians will have the right to access the final trial dataset.

### Statistical analysis

Statistical analysis will be performed by trial statisticians who are blinded to the group allocation using SPSS Statistics Software 19.0 and SAS version 9.4. All statistical tests will be two sided with a significance level of 0.05. We will summarize the social demographic characteristics of the participants by groups using simple descriptive statistics. Normally distributed quantitative variables will be described by means and standard deviation and non-normally distributed variables will be described by median and interquartile range. In addition, the 95% confidence intervals will be presented when calculating the difference in means between groups. For categorical variables, numbers and percentages will be presented.

We intend to use the intention-to-treat and per-protocol approach to analyze data. For intention-to-treat analysis, all participants will be included in the analysis according to the group to which they were originally randomized, regardless of whether they complete the treatment or adhere to the protocol. For per-protocol analysis, only participants who complete the whole treatment (i.e., those who receive treatments once per day for 3 consecutive days) will be included. Between-group comparisons will be performed using two-sample *t* tests for normally distributed data and Mann–Whitney *U* test for non-normally distributed data. For categorical variables, chi-square tests or Fisher’s exact test will be used for group comparison.

Multivariable regression analysis will be performed to analyze the associations between pediatric *Tuina* and primary or secondary outcomes with adjustment for covariates including social demographic characteristics, birth characteristics, medication history, and so forth. Missing data will be imputed through multiple imputation using chained equations.

### Adverse event management

During the study period, all adverse events experienced by the participant throughout the treatment and follow-up periods, regardless of relevance to the interventions, will be documented and immediately reported to the Ethics Committee. The time of occurrence, severity, duration and treatment of AEs will be documented on the AE report form and dealt with using appropriate treatment methods. Potential AEs include local skin damage and rash. The Principle Investigator will supervise the handing of AEs and ensure that adequate action has been taken.

## Ethics and dissemination

### Protocol amendments

Any important protocol amendments will be submitted to the Ethics Committee for approval. After approval, an update will be made to ClinicalTrials.gov.

### Consent or assent

The study has been approved by both Ethics Committees based in Guangdong Provincial Hospital of Chinese Medicine as well as in Dongguan Kanghua Hospital. Written informed consent will be obtained from the guardians or parents of all trial participants by trained investigators. Additionally, oral informed consent will also be obtained when the children are between 3 and 6 years of age. The investigators will explain the nature of the study, purpose, potential risks and benefits in detail to each participant and their guardians or parents. After the participants’ guardians have read and understood the nature of the study, they will be provided with adequate time to decide whether or not to participate. The participants and their guardians have the right to refuse to join the study or to withdraw from the study at any time during the study. Their decision will not affect subsequent medical assistance and treatment.

### Ancillary and post-trial care

If patients experience adverse events during the trial, a medical expert committee will determine if it is related to the trial. The medical expert committee includes members from the Ethics Committee of Guangdong Provincial Hospital of Chinese Medicine, members from the Ethics Committee of Dongguan Kanghua Hospital, and the Principle Investigator. Researchers will provide the cost of treatment and financial compensation for experiment-related harm according to guidance from the Good Clinical Practice Act.

### Dissemination policy

Both positive and negative trial results will be disseminated in several ways. They will be presented at key international conferences, published in peer-reviewed and high-ranking journals, made available on specialist websites, disseminated by the Pediatric *Tuina* Committee, and provided as feedback to patients or professionals by holding *Tuina* workshops or seminars.

## Discussion

The current study is designed to evaluate the efficacy of pediatric *Tuina* for children with acute diarrhea. A series of trial feasibility criteria have been established. If all criteria are met, the trial will be considered feasible. If they are not met, and revisions are not possible, the trial will be considered unfeasible. Where the trial is considered unfeasible, and revisions are possible, details of suggested revisions will be described in feasibility trial outputs. Feasibility criteria have been established as follows: 1) recruitment is feasible, indicated by randomization of the targeted population; 2) treatment and attendance are acceptable, indicated by attending more than 67% of the anticipated three sessions offered; 3) data collection procedures are feasible, indicated by sessions recorded; 4) missing data are at an acceptable level, indicated by 80% of visits completed and minimal noncompletion of within-visit questionnaires; and 5) estimates needed for sample size calculation are obtainable.

We anticipate two main challenges to be overcome in this study. The first challenge is how to choose a reasonable comparison. In previous studies, researchers often chose the following interventions for the control group [31–35]: 1) standard/usual care; 2) sham/placebo massage, meaning gentle, still touch producing no indentation in the skin; 3) therapeutic touch from the west; 4) special TCM treatments, including acupuncture, Chinese herbal medicine, cupping and so on; 5) blank control; and 6) other controls. In clinical trials based on evidence-based medicine, validation of treatment modalities has invariably adopted the procedures of randomization and placebo-controlled designs [36]. However, the development of placebo controls has been a unique challenge in evaluating the specific benefits of *Tuina*. Ideally, to exclude the potential placebo effect, participants should not know whether they are receiving true or sham intervention treatment in double-blind, controlled clinical trials [37].

In recent years, some researchers have used sham massage (gentle, still touch producing no indentation in the skin) as control treatment in clinical trials and showed that this gentle, still touch was ineffective [38]. Although this method improves the quality of study, there are still some disadvantages for older children who have previously experienced pediatric *Tuina*, particularly in clinical trials of blind design. Specifically, those children and their guardians who are familiar with *Tuina* may be skeptical about the manipulation received and hinder the validity of blinding. In our study, we use a cloak-shaped device to cover the upper body and arms of the child. Sham *Tuina* manipulation consists of the therapist using one hand to hold the child’s hand or putting one hand on the child’s body while the other hand performs manipulations under the cloak on the therapist’s own hand instead of the child’s hand or body. In addition, we only include children who are under 6 years old and exclude children who have a history of experiencing Chinese pediatric *Tuina* on the hands. Therefore, this design can avoid previous limitations in other research.

The second challenge is the implementation of the blinding method. The use of blinding is regarded as an important criterion to evaluate the methodological quality of clinical trials [39]. Due to the characteristics of pediatric *Tuina* and the particularity of operation, it is difficult to design double-blind clinical trials. Therefore, most studies are designed as open trials. Although a few studies used the blind method in clinical trials, treatment assignment was only masked to the outcome assessor and/or investigator. Some studies made use of a privacy screen, where massage was performed behind the screen to maintain “masking” of the infant’s group assignment to parents and newborn intensive care unit clinical staff [40]. However, *Tuina* can be difficult to perform on younger children without the presence of their guardian. We designed a cloak-shaped device to blind the children’s guardians. The cloak covers the child’s hands and upper body and all manipulations will be performed under the cloak. As a result, guardians can be blinded and present during treatment. This type of blinding can make up for the deficiency of previous studies.

To reduce possible bias and improve the quality of study, the following methods will be adopted. First, this study is designed as a randomized, double-blinded trial. Randomized methods and allocation concealment will be performed according to a standardized procedure. Except for the *Tuina* therapists, all patients, caregivers, study staff, outcome assessors and data statisticians will be masked. Second, this study will use a sham *Tuina* method as a control and a specifically designed cloak-shaped device to cover the upper body of a child for the purpose of blinding. This method is pioneering in a clinical study. Third, *Tuina* therapists, data collectors and outcome assessors will participate in training before the trial commences. All operating procedures including *Tuina* manipulation, acupoint selection and outcome assessment will be carried out strictly according to the standardized methods.

In conclusion, this protocol provides a standardized process to guide subsequent clinical research. We expect the trial to demonstrate the efficacy of *Tuina* therapy for the treatment of acute diarrhea. The results of this trial will help doctors have a deeper understanding of *Tuina* therapy and will provide valuable evidence for future research in this area.

## Trial status

The protocol was registered on 29 December 2016. The first participant was recruited into the trial on 8 January 2017. Recruitment will be completed in September 2019. The current protocol is version 4.0, 30 April 2019.

## Supplementary information

**Additional file 1.** SPIRIT 2013 checklist.

## Data Availability

The results of this trial will be presented in peer-reviewed journals.

## Abbreviations

AE: Adverse event
CRF: Case report form
GCR: Global change rating
ORS: Oral rehydration solution
RCT: Randomized controlled trial
SPIRIT: Standard Protocol Items Recommendations for Interventional Trials
TCM: Traditional Chinese medicine
WHO: World Health Organization
